# Correlation of GNAS Mutational Status with Oncologic Outcomes in Patients with Resected Intraductal Papillary Mucinous Neoplasms

**DOI:** 10.3390/cancers17040705

**Published:** 2025-02-19

**Authors:** Julia Evans, Kylee Shivok, Hui Hsuan Chen, Eliyahu Gorgov, Wilbur B. Bowne, Aditi Jain, Harish Lavu, Charles J. Yeo, Avinoam Nevler

**Affiliations:** 1 Sidney Kimmel Medical College, Philadelphia, PA 19107, USA; julia.evans2@students.jefferson.edu (J.E.); kylee.shivok@students.jefferson.edu (K.S.); hui.chen@students.jefferson.edu (H.H.C.); 2 Jefferson Pancreas, Biliary and Related Cancer Center, Sidney Kimmel Cancer Center, Philadelphia, PA 19107, USAcharles.yeo@jefferson.edu (C.J.Y.)

**Keywords:** IPMN, intraductal papillary mucinous neoplasm, IPMN-associated pancreatic cancer, oncologic outcomes, pancreatic resection, GNAS, KRAS, P53

## Abstract

**Background:** Intraductal papillary mucinous neoplasms (IPMNs) are pre-malignant pancreatic lesions that may progress to invasive pancreatic ductal adenocarcinoma (PDAC). IPMN-associated invasive carcinoma (iIPMN) has been associated with more favorable survival outcomes compared to non-iIPMN-derived PDAC. Here, we aim to investigate the genetic landscape of IPMNs to assess their relevance to oncologic outcomes. **Methods:** This retrospective study used a large single-institution prospectively maintained database. Patients who underwent curative-intent pancreatic resection between 2016 and 2022 with histologically confirmed diagnosis of IPMN were included. Demographic, pathologic, molecular, and oncologic outcome data were recorded. Kaplan–Meier survival analyses were performed. PDAC data from public genetic databases were used for mutational correlation analysis. *p*-value ≤ 0.05 was considered as significant. **Results:** A total of thirty-nine patients with resected IPMN with complete clinical and sequencing data were identified and included in the final cohort. The male-to-female distribution was 21:18, and the mean age was 70.1 ± 9.1 years. GNAS mutations occurred in 23.1% of patients, and 89.7% of patients had iIPMN. In iIPMN patients, GNAS mutation was strongly associated with improved disease-free survival: all GNAS-mutant patients survived to follow-up with significantly fewer recurrences than in GNAS wild-type (WT) patients (*p* = 0.013). Mutated GNAS closely co-occurred with wild-type KRAS (*p* < 0.001), and further analysis of large genomic PDAC datasets validated this finding (OR 3.47, *p* < 0.0001). **Conclusions:** Our study suggests prognostic value of mutational status in malignant resected IPMNs. WT GNAS, mutant P53, and mutant KRAS each correlate with recurrence and decreased survival. Further studies are required to validate these preliminary observations.

## Simple Summary

Pancreatic ductal adenocarcinoma arising from intraductal papillary mucinous neoplasms (IPMNs) has been observed to be associated with improved survival outcomes compared to other pancreatic ductal adenocarcinomas. Using a next gene sequencing panel, we analyzed resected IPMNs and correlated their mutational status with oncologic outcomes. Our analysis revealed a strong correlation between GNAS mutations, non-mutated KRAS status, and improved overall and recurrence-free survival.

## 1. Introduction

Intraductal papillary mucinous neoplasms (IPMNs) are a common, pre-malignant type of pancreatic cystic lesion [1]. With recent advances and increased frequency of imaging, more incidental pancreatic cysts are being identified in asymptomatic patients [2,3]. The prevalence of incidentally-found IPMNs in patients 50 years or older is approximately 10%, most of which are considered low-risk and of the branch-duct (side-branch) type [4]. However, IPMNs carry an increased risk of malignant transformation, estimates of which amount to 22% of resected branch-duct IPMNs [5] and 55–60% of resected main-duct IPMNs [6,7].

Approximately 19% of resectable pancreatic cancers originate from IPMNs, and in patients with high-risk IPMN, the incidence of pancreatic cancer is 34 cases per 100 persons compared with 0.11 cases per 100 persons in patients without IPMN [4]. However, despite their classification, IPMN-related invasive pancreatic ductal adenocarcinomas (iIPMN) have been reported to carry a better prognosis than pancreatic ductal adenocarcinomas (PDACs) of any etiology, with 5-year post-resection survival rates of 38% and 17%, respectively [8,9].

Genetic understanding of IPMNs has greatly advanced in the past decade. GNAS and RNF43 mutations have been found to be specific markers for IPMN and IPMN-associated PDAC [10,11,12,13]. The combined utilization of GNAS and KRAS mutations as tumor markers has allowed for improved preoperative detection of IPMN; mutation in one or both of these genes has been observed in up to 96% of IPMN cases [12], with high sensitivity and specificity for IPMNs of approximately 79% and 98%, respectively [14]. In addition, GNAS mutations have been found to correlate strongly with the intestinal subtype of IPMN [11,15,16,17], which carries a more favorable prognosis. The importance of these findings lies in their impact on patient outcomes.

The process of malignant transformation of IPMNs is still poorly understood. Mutations in the oncogenes GNAS and KRAS are believed to be the most prevalent driver mutations in IPMN development [18]. Multiple pathways of progression have been proposed due to the multifocal nature of IPMNs and evidence that these lesions develop from polyclonal evolution [18,19]. The mutational status of GNAS and KRAS, among other genes such as P53, may be used to predict prognosis and future malignant transformation [12,16].

Therefore, in this study, we aim to investigate the genetic landscape of IPMNs, and mutated GNAS/KRAS IPMNs specifically, to assess relevance with oncologic outcomes.

## 2. Materials and Methods

### 2.1. Study Population

This is a retrospective case–control study from a single high-volume, NCI-comprehensive cancer center. Data from a prospectively maintained pancreatic surgery database were reviewed to identify patients who underwent resection for a histologically confirmed diagnosis of IPMN or IPMN with invasive adenocarcinoma at our institution between January 2016 and December 2022. Patient outcomes were followed up to November 2024 using their electronic medical records. Only patients with full next-generation sequencing molecular analysis of the neoplastic genome were included in this study.

### 2.2. Data Collection

Demographic, perioperative, pathologic, molecular, and oncologic outcome data were consolidated through a single-institution, large, prospectively maintained database. Patients with a mutant form of GNAS were differentiated from patients with wild-type GNAS status for comparison of within-group characteristics. Overall, a total of 194 records of patients with IPMN were reviewed. Due to the relative paucity of next-generation sequencing testing performed, specifically of benign lesions, a total of 39 IPMN records with complete molecular data were identified and included in the final study cohort. Thirty-five of these records involved IPMN-PDAC and thus were analyzed as a subgroup to account for differences between IPMNs with high malignant potential and the majority of IPMNs with low likelihood of transformation.

### 2.3. Bioinformatics

Molecular data from the Tumor Cancer Genome Atlas (TCGA), Clinical Proteomic Tumor Analysis Consortium (CPTAC), Memorial Sloan Kettering (MSK), and the International Cancer Genome Consortium (ICGC) pancreatic cancer cohorts were queried through the cBioPortal platform (https://www.cbioportal.org/ (accessed on 26 November 2024)). A total of 2759 tumors were identified. GNAS and KRAS mutation data were recorded and analyzed for statistical associations.

### 2.4. Statistical Analysis

Nominal variables are expressed as numbers with percentages. Continuous variables are expressed as means (±SD). Student’s *t*-test and one-way ANOVA (with post-hoc Tukey HSD) were used to compare continuous variables. Survival status was calculated by univariable analysis using the Kaplan–Meier method, with log-rank testing for comparison of subgroups. Due to the small cohort size, multivariable regression analyses (e.g., Cox regression) were limited in the number of assessed covariates based on 1/10 of the sample size. *p* values ≤ 0.05 were considered to be statistically significant. Statistical data were analyzed with IBM SPSS (29.0.1.0, IBM, Armonk, NY, USA).

### 2.5. Ethical Review

This study was performed at Thomas Jefferson University Hospital (TJUH) and approved by the local institutional review board (Protocol #22E.136). The data supporting the findings of this study are available from the corresponding author upon request.

## 3. Results

### 3.1. Demographics and Clinical Characteristics

One hundred ninety-four patients with IPMNs were resected during the study period, including 127 pancreaticoduodenectomy procedures, 57 distal pancreatectomies, and 10 total pancreaticoduodenectomies. Of these, 39 evaluable patients with complete clinical and next-generation sequencing data were identified and included in the final cohort. The male-to-female distribution of the 39 patients was 21:18, with GNAS mutant IPMNs occurring in six males and three females. Of note, in the invasive IPMN subgroup, all patients with mutant GNAS lesions were male (*p* = 0.02). In the overall cohort, the mean age was 70.1 ± 9.1 years, similar to the mean age of 70.2 ± 9.3 years in the invasive cases. Of the 39 patients undergoing resection included in the final cohort, 35 had invasive IPMN-associated adenocarcinoma, with the remaining four cases presenting with various grades of dysplasia. Of the 35 patients with invasive IPMNs, 65.7% were main-duct IPMNs, 17.1% were side-branch IPMNs, and 17.1% were mixed main-duct and side-branch IPMNs. All of the invasive adenocarcinomas arose in association with IPMN lesions. KRAS mutations were identified in 85.7% of the patients with invasive IPMNs (43.3% G12D, 26.6% G12V, 26.6% G12R, 3.3% G12A, 3.3% Q61H, and 3.3% Q61R), and GNAS mutations were identified in 17.1% (66.7% R201C and 33.3% R201H). Various P53 mutations occurred in 51.4% of patients with invasive lesions. Further characteristics of patients are described in Table 1. The detailed demographics and characteristics of patients with IPMN-associated invasive adenocarcinoma are described in Table 2.

Significant differences in concurrent mutations were found between patients with wild-type GNAS (WT GNAS) and mutant GNAS invasive IPMNs. KRAS mutations were present more frequently in patients with WT GNAS than those with GNAS mutations in invasive IPMNs (OR 36.3, 95% CI 3.3–394.8, *p* < 0.001). Additionally, 62.1% of patients with GNAS WT had P53 mutations, compared with no patients in the GNAS mutation group (0%) (*p* = 0.006, Table 1). Concordantly, patients in the GNAS-WT group were more likely to present with perineural invasion compared with patients from the mutant GNAS group of invasive IPMNs (89.7% vs. 50%, *p* = 0.019).

### 3.2. Survival Outcomes

The median overall survival from the time of resection for the entire cohort (*n* = 39) over the study period was 67.3 months (±22.39 months), with a disease-free survival of 32.6 months (±16.10 months). In the IPMN-associated invasive adenocarcinoma group (*n* = 36), the median overall survival time was 56.8 months (±24.58 months), and the median disease-free survival was 24.9 months (±5.40 months).

### 3.3. Impact of GNAS, KRAS, and P53 Mutations on the Survival Outcomes of IPMN-Associated Invasive Adenocarcinoma

**Figure 1:**
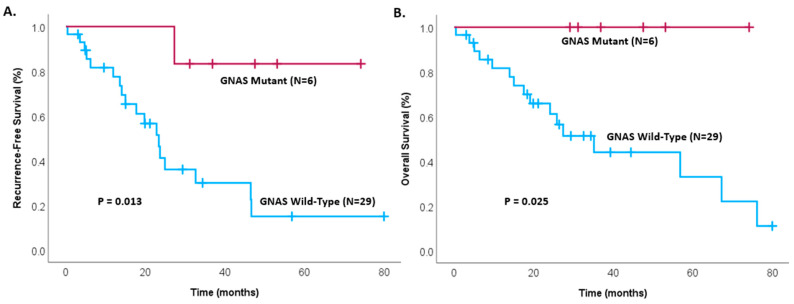
Kaplan–Meier survival analysis comparing patients with GNAS mutant invasive IPMNs and GNAS wild-type invasive IPMNs for (**A**) recurrence-free survival (*p* = 0.013) and (**B**) overall survival (*p* = 0.025) in patients after complete resection. IPMN—intraductal papillary mucinous neoplasm.

**Figure 2:**
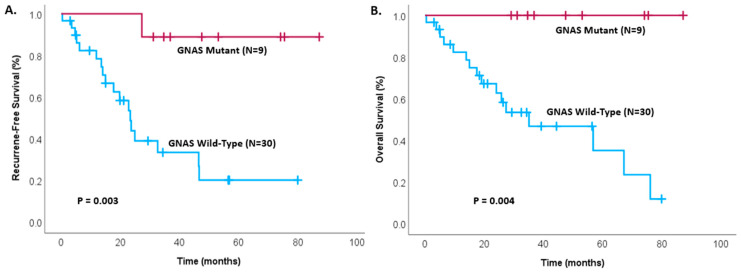
Kaplan–Meier survival analysis comparing all patients (invasive and non-invasive IPMNs) with GNAS mutant IPMNs and GNAS wild-type IPMNs for (**A**) recurrence-free survival (*p* = 0.003) and (**B**) overall survival (*p* = 0.004) in patients after complete resection.

Among the 35 patients with invasive IPMNs, disease-free survival was found to be significantly greater in GNAS mutant patients, with GNAS mutant patients not reaching their median survival, while GNAS wild-type patients had a median disease-free survival of only 23.4 months (Figure 1A, *p* = 0.013). Similarly, no deaths have occurred in patients with GNAS mutant status (*p* = 0.025), as seen in Figure 1B. A comparison of the impact of GNAS mutational status on the survival of the entire cohort, including all IPMN cases (invasive and non-invasive, *n* = 39), is shown in Figure 2.

**Figure 3:**
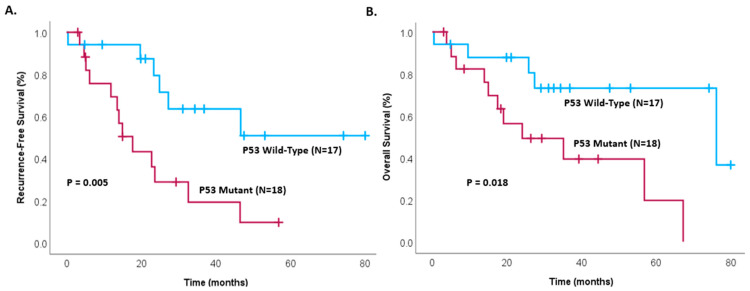
Kaplan–Meier survival analysis of patients with invasive IPMNs comparing patients with P53 mutant and P53 wild-type tumors, showing (**A**) recurrence-free survival (*p* = 0.005) and (**B**) overall survival (*p* = 0.018) in patients after complete resection.

**Figure 4:**
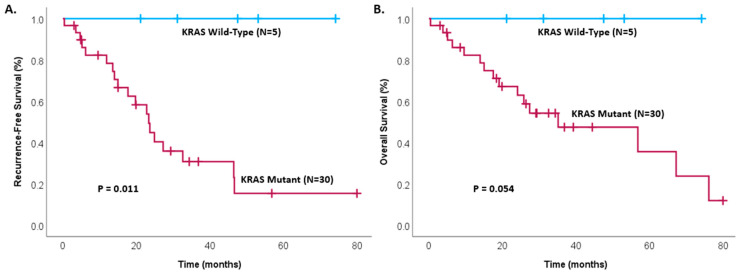
Kaplan–Meier survival analysis of patients with invasive IPMNs comparing patients with KRAS mutant and KRAS wild-type tumors, showing (**A**) recurrence-free survival (*p* = 0.011) and (**B**) overall survival (*p* = 0.054) in patients after complete resection.

The impact of other predictive genetic factors was also analyzed. Recurrence-free survival of patients with invasive IPMNs was found to be significantly greater in P53, with P53 wild-type patients not reaching their median survival, while P53 mutant patients had a median disease survival of only 17.7 months (*p* = 0.005). Patients with P53 wild-type status of invasive IPMNs also demonstrated significantly greater overall survival time (76.2 months vs. 24.1 months, *p* = 0.018), as shown in Figure 3. No patients with KRAS wild-type invasive IPMNs have experienced death, and there is significantly lower recurrence and a trend toward lower overall survival time in KRAS wild-type patients compared to KRAS mutant patients (*p* = 0.011 and *p* = 0.054, respectively), as shown in Figure 4.

Cox multivariable survival models were used to assess the impact of GNAS, P53, and KRAS mutation status on survival outcomes. Overall survival models were analyzed, assessing T stage and N stage, and the individual mutations were unable to converge. Analysis of recurrence-free survival models resulted in several findings: in a KRAS-based model (including KRAS mutational status, T stage, and N stage, *p* = 0.02), none of the covariates were found to be independently significant; in a P53-based model (*p* = 0.036), T stage and N stage were not found to be significant covariates, while P53 mutational status was found to be a prognostic risk factor (HR 4.2, 95% CI 11.7–1.5, *p* = 0.006); and in a GNAS-based model (*p* = 0.065), T stage and N stage were not found to be significant covariates, while GNAS wild-type status was found to be a prognostic risk factor (HR 10.1, 95% CI 1.3–78.6, *p* = 0.027).

### 3.4. Interaction Between KRAS Mutations and GNAS Mutations in IPMN and PDAC

As our study revealed that GNAS mutations were almost mutually exclusive of KRAS mutations, we decided to further investigate the relationship between KRAS and GNAS using previously published datasets and available large open molecular databases.

We first analyzed the data of Wu et al. [12] detailing specific GNAS and KRAS mutation status in IPMN samples (cyst fluid and micro-dissected cyst wall specimens) of 132 patients. Examination of the data showed an enrichment of KRAS wild-type status in cysts with GNAS mutation, though this was not found to be statistically significant (OR 2.3, CI 95%: 0.7–7.3, *p* = 0.1).

Next, we queried four databases through the cBioPortal platform (MSK-IMPACT, CTPAC, TCGA, ICGC) and reviewed GNAS and KRAS mutation data of 2759 pancreatic cancer patients. The mean age of PDAC diagnosis of GNAS -mutant patients was older than GNAS wild-type patients (67.8 ± 11.4 vs. 65.4 ± 10.5, respectively. *p* < 0.05). GNAS mutant patients were more likely to present with a resectable disease rather than locally advanced or metastatic pancreatic cancer (OR 1.8, 95% CI 1.1–2.8, *p* < 0.05). Analysis of GNAS and KRAS mutation incidence in this cohort revealed that GNAS mutations were strongly associated with the co-occurrence of a KRAS wild-type genotype (OR 3.47, 95% CI: 2.09–5.74, *p* < 0.0001).

## 4. Discussion

IPMNs are common pancreatic cystic lesions that can undergo malignant degeneration and, therefore, hold a significant risk for cancer. Both noninvasive cystic lesions, like IPMN, and noninvasive, non-cystic lesions, like pancreatic intraepithelial neoplasia (PanIN), may progress to PDAC. However, IPMN-associated PDACs express a different genetic landscape compared with PanIN-derived PDACs and also result in different oncologic outcomes. This current study aimed to investigate potential genetic prognostic factors to further elucidate these disparate outcomes.

Our study found that GNAS mutant IPMN lesions were relatively enriched with wild-type KRAS and P53 genotypes. These GNAS-mutant lesions tended to be of lower grade and less invasive. In invasive IPMNs, GNAS-mutant lesions correlated with improved survival outcomes. Further analysis of molecular data from additional IPMN and PDAC datasets supported an inverse association between GNAS mutation and KRAS mutations.

Tulla et al. identified multiple genetic markers that may hold prognostic value for patients with IPMNs, including the oncogenes KRAS, GNAS, and BRAF, along with tumor suppressor genes such as P53, CDKN2A, and SMAD4 [20]. Wu et al. proposed that mutations in GNAS can provide a unique and parallel mechanism for IPMN progression, independent of the more established mechanism known to involve KRAS mutations, evidenced by tumors with GNAS mutant status lacking mutant KRAS [12] and vice versa. This was further supported by their analysis of invasive IPMN tumors, showing matching mutational profiles to the surrounding, non-invasive, dysplastic IPMNs. Further data can be gleaned from the study of Tan et al. [21], who reported the mutational analysis of invasive and non-invasive IPMN, which noted frequent mutations in KRAS, GNAS, P53, and RNF 43. While KRAS and GNAS mutations appeared in similar proportions in low-grade, high-grade, and invasive lesions, mutations in P53, RNF43, SMAD4, MLL2, ATM, CDKN2A, ATM, and BRAF appeared in higher-grade lesions.

The oncologic outcomes of patients with resected IPMNs can vary greatly based on factors such as histopathology and degree of invasion. Histologically, IPMNs have been classified into three identifiable subtypes: intestinal, pancreaticobiliary, and gastric. Of these, the pancreaticobiliary type has been associated with a greater risk of invasion and poorer survival than the other subtypes, while the intestinal subtype has been found to have significantly better survival [22,23,24]. This is likely due to the strong correlation between colloid carcinoma and the intestinal subtype of IPMNs, as IPMN-associated colloid carcinoma carries a more favorable prognosis than IPMN-associated PDAC [8,17,25]. In a previous meta-analysis, Lee et al. found a significant correlation between GNAS mutations and the intestinal subtype, with a high frequency of GNAS mutations found in 74% of intestinal-type IPMNs [16]. Hata et al. further established this relationship by finding GNAS mutations in cfDNA of primary IPMN lesions to be associated with the acellular hypersecreting mucin pools lacking neoplastic epithelium in intestinal-type IPMNs [11]. Although our present study was not able to distinguish histologic subtypes of each case, the correlations between the intestinal subtype with GNAS mutations and improved prognosis support our findings regarding GNAS mutations and greater survival time. The differences in GNAS and KRAS mutational status and unique pathological behavior observed in the intestinal subtype support the potential driver role of GNAS in histopathological tumor development with unique outcomes that may promote GNAS status as an early prognostic marker itself.

Tumor invasion has also been studied in an attempt to predict outcomes for patients with IPMNs. Marchegiani et al. reviewed 412 patients with IPMN resections and observed a five-year survival rate of 82% after resection of IPMNs, with cancer recurrence rates of 45% and 9% for invasive and noninvasive cases, respectively [26]. In our cohort, over the observed study period of 6 years, we found a survival rate of 55%, with all deaths occurring in invasive cases. Similarly to Marchegiani et al., recurrence rates in our cohort were 40% and 0% for invasive and noninvasive cases, respectively. Our study took this analysis a step further by determining that recurrence and survival also correlated with GNAS/KRAS/P53 mutant status.

We aimed to study invasive and non-invasive IPMNs and compare their outcomes based on the expression of GNAS, KRAS, and P53 mutations. Our study found that patients with wild-type P53, wild-type KRAS, or GNAS mutations have markedly better overall and disease-free survival. While KRAS and P53 are known prognostic factors in pancreatic cancer survival, the prognostic value of GNAS in iIPMN merits further discussion: Gaujoux et al. found GNAS mutations to be associated with improved survival in 31 cases of resected invasive IPMNs [17]. Asano et al. similarly found mutant GNAS status to be correlated with improved overall survival in patients with resected high-grade and invasive IPMNs (*n* = 18) [27]. Unfortunately, their small sample size likely prevented the employment of a multivariable regression model to determine the full extent of the impact of GNAS mutations. Similarly, Tan et al. [21], in their assessment of 38 invasive IPMNs, noted favorable survival in GNAS-mutant patients but were unable to use a multivariable regression model due to their limited sample size. Interestingly, in their study, GNAS mutation outperformed KRAS mutation as a prognostic factor. These multiple observations supporting a possible association of GNAS mutation with a favorable prognosis in iIPMN may be explained, in part, by the association of GNAS mutation with decreased tumor invasion—as seen in our study, as well as in the studies of Gaujoux et al. and Ideno et al., which showed invasive IPMNs to be enriched with wild-type GNAS and only 11–25% of invasive IPMNs to have GNAS mutant status [17,28]. However, larger cohorts are needed to fully uncover the independent impact of GNAS mutation status on survival. Overall, our study corroborates these previous small-scale studies by validating that GNAS mutant status is associated with improved overall survival and lower rates of recurrence.

KRAS mutations were identified in the majority of our IPMN cohort, including in the invasive IPMN cases. Other studies have demonstrated KRAS mutations in 36–81% of IPMN cases [29], which is slightly lower than our incidence, possibly due to the majority of our cohort having invasive progression. KRAS mutations are found in over 90% of conventional pancreatic adenocarcinomas, indicating that the KRAS pathway is heavily associated with invasion, whereas GNAS mutations are less commonly identified [17,21]. Interestingly, in the study published by Hata et al., identifying mutations in cfDNA of primary IPMNs, KRAS mutations were found in a mere 6% of biopsied lesions [11]. This brings into question the different roles GNAS and KRAS mutations play in the biological spectrum of IPMN development. We have also shown that patients with mutant GNAS status have significantly lower rates of concurrent KRAS mutations. A similar finding can be seen upon analyzing data from Wu et al. [12], though the effect does not reach statistical significance. As KRAS mutation status is a major determinant of survival in PDAC, we sought to generalize the scope of this association by looking at a large set of PDAC samples and finding a very similar association. This corresponds with the recent work of Takebe et al., which found GNAS mutations and mutations in Wnt pathway signaling genes (RNF43, CTNNB1, and APC) to be associated with improved overall survival in patients with metastatic PDAC [30]. Of these genes, GNAS and RNF43 are both frequently mutated in iIPMN.

As KRAS and P53 mutations were frequent in our cohort and were found to be associated with survival, it is hard to accurately determine the individual impact of these mutations on survival. Both KRAS and P53 mutations have been shown to be strong prognostic markers for pancreatic cancer survival. Since GNAS mutations have been shown to occur early on in the neoplastic progression of IPMN, while P53 mutations tend to be associated with more high-grade or invasive lesions [21], it is possible that GNAS and KRAS statuses may be more appropriate for use as factors, from a chronological progression viewpoint. As we observed KRAS mutations to closely associate with wild-type GNAS IPMN lesions, we decided to use a broader cohort of PDAC cases, not uniquely limited to IPMN, to test whether this association is relevant in a more general context in pancreatic cancer. Our analysis showed that pancreatic adenocarcinoma specimens with wild-type KRAS status were 3.5 times more likely to have a GNAS mutation. This finding is intriguing and merits further research.

Only one patient in our cohort was found to have a mutation in neither GNAS nor KRAS genes. Instead, this patient had a loss of SMAD4 expression, with a stop codon at amino acid Q245. Although this patient’s pathology revealed invasive adenocarcinoma arising from an IPMN, this lesion has not recurred, nor has this patient died. This supports previous work indicating that SMAD4 mutation does not necessarily co-occur in progression with KRAS mutations [31]. While KRAS and P53 mutations are fairly common mutations in PDAC and in iIPMN, GNAS appears to be more specific to iIPMN. Guanine Nucleotide binding protein, Alpha Stimulating activity polypeptide (GNAS) codes for the alpha subunit of a G-protein-coupled receptor, and mutations often result in overactivation of the G-protein pathway, with increased levels of cyclic AMP [12,13]. Due to this ‘pro-oncogenic’ role of GNAS mutations, the data suggesting its association with improved survival may seem puzzling. However, as GNAS mutations act as a separate pathway to the KRAS neoplastic progression, and our data suggest that KRAS wild-type occurs more frequently in GNAS mutants, it is possible that GNAS mutations are a weaker oncogenic driver than KRAS mutations.

There are a few limitations that need to be considered in the present study. First, the small sample of cases, and especially the few cases with GNAS mutations present, clearly limits the statistical power and ability to infer clear conclusions from the data obtained. The size also limits our ability to correct for confounding variables with survival regression models, as seen by the limited results in our regression analysis. Second, despite our use of a prospectively maintained database, the study is still, in nature, retrospective and thus susceptible to selection and misclassification biases. Although we have shown an association between GNAS mutation and improved survival outcomes in PDAC, the mechanism for this has not been elucidated. Additionally, although our cohort includes patients with both malignant and benign IPMNs, we have performed a separate, dedicated subgroup analysis including only invasive IPMNs, and the impact of GNAS in the entire cohort and this subgroup must be considered in this context. Moreover, the cohort only represents resected IPMNs; thus, it is not fully reflective of all IPMN cases.

The management algorithm of IPMNs is continuously evolving, from the 2006 Sendai guidelines to the 2012 Fukuoka guidelines and then to the 2017 revised Fukuoka guidelines, through to the 2018 European guidelines and the recently published 2024 Kyoto guidelines [32]. As we see the adoption of precision medicine approaches in other gastrointestinal domains, it would be interesting to see how and when such consideration will be added to the guidelines for the management of IPMN. The recent Kyoto guidelines state that the IPMN subtype can offer information in addition to the grade of dysplasia in clinical decision making. Will GNAS and other genetic markers be next? Further investigation in the upcoming years of the genetic landscape of IPMNs and the associated clinical outcomes will hopefully provide us with answers.

## 5. Conclusions

Our study suggests that IPMNs with GNAS mutations are associated with favorable overall and disease-free survival. IPMNs without GNAS mutations were associated with higher rates of KRAS and P53 mutations and an overall invasive histological phenotype. While these results highlight the potential utility of GNAS and other genetic markers to serve in clinical decision-making for IPMN, prospective clinical studies are required in order to validate these current observations.

## Figures and Tables

**Table 1:** Demographics and clinical characteristics of patients with invasive IPMN with regards to GNAS mutational status. BMI—Body mass index, IPMN—intraductal papillary mucinous neoplasm.

Characteristic	Overall Cohort (*n* = 35)	GNAS WT (*n* = 29)	GNAS Mutant (*n* = 6)	*p*-Value
**Age, years ± SD**	70.2 ± 9.3	71.5 ± 8.8	64.3 ± 10.2	0.09
**Sex**				0.02
Male	20 (57.1%)	14 (42.9%)	6 (100%)	
Female	15 (42.9%)	15 (57.1%)	0 (0%)	
**Preoperative BMI (kg/m^2^)**	25.5 ± 4.6	25.8 ± 5.0	24.1 ± 1.0	0.53
**Race**				0.404
Caucasian	28 (80%)	22 (75.9%)	6 (100%)	
African American	6 (17.1%)	6 (20.7%)	0 (0%)	
Asian	1 (2.9%)	1 (3.4%)	0 (0%)	
**Any smoking history**	17 (48.6%)	13 (44.8%)	4 (66.7%)	0.33
**Diabetes** **(>1 year prior to IPMN diagnosis)**	9 (25.7%)	6 (20.7%)	3 (50%)	0.135
**Any history of cancer**	11 (31.4%)	10 (34.5%)	1 (16.7%)	0.392
**Family history of cancer**	23 (65.7%)	19 (65.5%)	4 (66.7%)	0.957
**IPMN Duct Type**				0.789
Main Duct (MD-IPMN)	19 (54.3%)	15 (51.7%)	4 (66.7%)	
Branch Duct (BD-IPMN)	7 (20%)	6 (20.7%)	1 (16.7%)	
Mixed-Type IPMN	9 (25.7%)	8 (27.6%)	1 (16.7%)	
**PD Diameter of MD-IPMN, mm ±SD**	7.2 ± 3.9	6.7 ± 3.6	8.6 ± 4.8	0.372
**Cyst size of non-MD-IPMN**				0.192
<4 cm	20 (57.1%)	18 (62.1%)	2 (28.6%)	
≥4 cm	14 (40%)	10 (34.5%)	4 (66.7%)	
**Perineural invasion**	29 (82.9%)	26 (89.7%)	3 (50%)	0.019
**Lymphovascular invasion**	11 (31.4%)	11 (37.9%)	1 (14.3%)	0.318
**Tumor Size**				0.695
T1	10 (28.6%)	9 (31.0%)	1 (16.7%)	
T2	7 (20%)	6 (20.7%)	1 (16.7%)	
T3	18 (51.4%)	14 (48.3%)	4 (66.7%)	
Tx	0 (0%)	0 (0%)	0 (0%)	
**Nodal stage**				0.748
N0	18 (51.4%)	15 (51.7%)	3 (50%)	
N1	9 (25.7%)	8 (27.6%)	1 (16.7%)	
N2	8 (22.9%)	6 (20.7%)	2 (33.3%)	
Nx	0 (0%)	0 (0%)	0%	
**KRAS mutation present**	30 (85.7%)	28 (96.6%)	2 (33.3%)	<0.001
**P53 mutation present**	18 (51.4%)	18 (62.1%)	0 (0%)	0.006
**Any recurrence**	14 (40%)	14 (48.3%)	0	0.028

**Table 2:** Demographics and clinical characteristics of all patients with resected IPMNs with regards to GNAS mutational status. BMI—Body mass index, IPMN—intraductal papillary mucinous neoplasm.

Characteristic	Overall Cohort (*n* = 39)	GNAS WT (*n* =30)	GNAS Mutant (*n* = 9)	*p*-Value
**Age, years ± SD**	70.1 ± 9.1	71.0 ± 9.0	67.0 ± 9.2	0.255
**Sex**				0.379
Male	21 (54%)	15 (50%)	6 (67.7%)	
Female	18 (46%)	15 (50%)	3 (33.3%)	
**Preoperative BMI (kg/m^2^)**	26.1 ± 4.5	25.9 ± 4.9	26.5 ± 3.3	0.77
**Race**				0.278
White/Caucasian	32 (82%)	23 (76.7%)	9 (100%)	
Black/African American	6 (15.4%)	6 (20%)	0 (0%)	
Asian	1 (2.6%)	1 (3.3%)	0 (0%)	
**Any smoking history**	19 (48.7%)	13 (43.3%)	6 (66.7%)	0.219
**Diabetes** **(>1 year prior to IPMN diagnosis)**	10 (25.6%)	7 (23.3%)	3 (33.3%)	0.547
**Any history of cancer**	12 (30.8%)	11 (36.7%)	1 (11.1%)	0.145
**Family history of cancer**	25 (64.1%)	19 (63.3%)	6 (66.7%)	0.855
**IPMN Duct Type**				0.89
Main Duct (MD-IPMN)	20 (51.3%)	16 (53.3%)	4 (44.4%)	
Branch Duct (BD-IPMN)	8 (20.5%)	6 (20%)	2 (22.2%)	
Mixed-Type IPMN	11 (28.2%)	8 (26.7%)	3 (33.3%)	
**MPD Diameter of MD-IPMN, mm ± SD**	7.2 ± 3.8	6.8 ± 3.4	8.6 ± 4.8	0.359
**Cyst size for non-MD-IPMN (*n* = 16)**				0.588
<4 cm	24 (61.5%)	19 (63.3%)	5 (55.6%)	
≥4 cm	14 (35.9%)	10 (33.3%)	4 (44.4%)	
**Dysplasia grade**				0.042
Low-grade	2 (5.1%)	1 (3.3%)	1 (11.1%)	
Moderate-grade	1 (2.6%)	0 (0%)	1 (11.1%)	
High-grade	1 (2.6%)	0 (0%)	1 (11.1%)	
Invasive carcinoma	35 (89.7%)	29 (96.7%)	6 (66.7%)	
**KRAS mutation present**	33 (84.6%)	29 (96.7%)	4 (44.4%)	<0.001
**P53 mutation present**	18 (45%)	18 (60%)	0 (0%)	0.002
**Any recurrence**	14 (35.9%)	14 (46.7%)	0 (0%)	0.01

## Data Availability

The raw data supporting the conclusions of this article will be made available by the authors on request.

## Abbreviations

The following abbreviations are used in this manuscript:

IPMN	Intraductal papillary mucinous neoplasm
iIPMN	Invasive IPMN
PDAC	Pancreatic Ductal Adenocarcinoma
